# Beta-lactam and CMV induced drug hypersensitivity syndrome

**DOI:** 10.1186/2045-7022-4-S3-P80

**Published:** 2014-07-18

**Authors:** Simona Kasinskaite, Kristina Bliudziute, Ingrida Maciulaityte, Violeta Kvedariene

**Affiliations:** 1 Vilnius University, Lithuania; 2 Vilnius University, Center of Internal Medicine, Lithuania; 3 Vilnius University, Center of Pulmonology and Allergology, Lithuania

## 

Beta-lactam antibiotics are one of the most common causes of drug allergic reactions. These reactions may be manifested by a concomitant viral infections caused by HIV, CMV, HHV6 or EBV, especially in children and less common in adults.

The aim of our study is to present the case of adult patient with drug hypersensitivity syndrome caused by beta-lactams and manifestation of viral infection.

A 45 years female was hospitalized in Vilnius University Hospital Santariskiu Klinikos with a fever over 38.5°C, dry cought and generalized maculopapular rash with diffuse itchiness. One week before she took amoxicilline for seven days. The symptoms appeared 24 hours after the last dose of antibiotic intake. The elevation of liver enzymes was found. A blood test was without eosinophilia. CMV-G and CMV-M were positive. Diagnosis of drug hypersensitivity syndrome induced by CMV infection and beta-lactam was made. Symptoms disappeared after a few weeks of treatment. Four months later drug provocation tests were performed. Patch test with amoxicillin was negative, as well as intradermal test with ampicillin, amoxicillin, penicillin G and cefuroxime. OPT with amoxicilin was without immediate reaction, however, generalized erythema appeared 7 hours later. Additionally, OPT with cefuroxime was without immediate reactions, however, generalized maculopapular rash and itchiness appeared 7-8 hours post OPT.

The present case is an example of adult patient with CMV induced acute infection and the manifestation of true allergic reaction to beta-lactam antibiotics at the same time.

